# Attitudes towards and experiences with sourdough and baker’s yeast bread amongst participants in a randomised controlled trial: a qualitative study

**DOI:** 10.29219/fnr.v66.8839

**Published:** 2022-10-21

**Authors:** Lisa Garnweidner-Holme, Marit Hallquist, Solveig Ivara Watters, Mia Gjøvik, Marius Pihl Frederiksen, Stephanie Jonassen, Ina Ravnanger, Christine Henriksen, Mari C.W. Myhrstad, Vibeke H. Telle-Hansen

**Affiliations:** 1 Department of Nursing and Health Promotion, Faculty of Health Sciences, Oslo Metropolitan University, Oslo, Norway;; 2 Mesterbakeren AS, Oslo, Norway;; 3 Department of Nutrition, Faculty of Medicine, Institute for Basic Medical Science, University of Oslo, Oslo, Norway

**Keywords:** sourdough bread, yeast bread, gastrointestinal symptoms, fibre, taste, qualitative study

## Abstract

**Background:**

Bread is an important source of dietary fibre. However, an increasing number of individuals exclude bread from their habitual diet for various reasons. In recent years, sourdough bread has increased in popularity, and clinical studies have indicated that sourdough bread may decrease gastrointestinal symptoms.

**Objective:**

To investigate attitudes towards and experiences with sourdough and baker’s yeast bread amongst participants in a randomised controlled trial (RCT), the health effects and consumer aspects of bread (HELFAB) study.

**Design:**

We conducted individual interviews with 10 participants who stated to be sceptical about bread and who participated in an RCT to investigate the health effects of sourdough bread versus baker’s yeast bread. The participants were interviewed on two occasions (before and after the RCT). Interviews were conducted digitally between September and December 2020 and were thematically analysed.

**Results:**

Half of the interviewed participants experienced gastrointestinal symptoms, such as pain in the stomach, when they consumed bread prior to the RCT. They often preferred sourdough bread to baker’s yeast bread both before and after the study, since they found that sourdough bread was easier to digest. Participants who were sceptical about bread prior to the study became more positive about bread because of their experiences with the intervention breads. This finding was mainly related to the taste and consistency of sourdough bread. The participants often associated bread with healthiness, mainly due to the dietary fibre content in bread.

**Conclusions:**

Sourdough bread with increased dietary fibre may be an important source of dietary fibre for those who perceive gastrointestinal problems from baker’s yeast bread. Participants in this qualitative study stated to change their attitudes towards bread, mainly due to perceived healthiness of the intervention bread.

## Popular scientific summary

• Many people exclude bread from their habitual diet. This qualitative study investigates reasons for excluding bread and experiences with bread during a randomised controlled trial (RCT).
• Participants who stated to be sceptical towards bread prior to participation in an RCT changed their attitudes, mainly due to the perceived healthiness of the intervention bread.
• Participants in this qualitative study preferred sourdough bread mainly related to the taste and consistency.
• Sourdough bread may be an appropriate alternative for yeast baked bread for individuals who experience gastrointestinal symptoms.

In many countries, bread is an important source of dietary fibre. Dietary fibre is known to reduce the risk of non-communicable diseases by improving blood lipids and glycaemic regulation, maintaining weight loss and beneficially affecting immune function (1, 2). However, fermentable oligosaccharides, disaccharides, monosaccharides and polyols (FODMAPs) have been linked to gastrointestinal ailments (3, 4). Bread may also be a source of salt in a habitual diet (5). Excess salt intake is a major contributor to hypertension and cardiovascular disease (6).

The intake of both bread and fibre does not meet the national recommendations in many countries, and there are indications that an increasing number of individuals exclude bread from their habitual diet (7). Carbohydrate restriction has become a popular diet for losing weight and regulating blood sugar (8). Furthermore, changes in consumers’ perception of bread quality, their preferences and the increase in gluten-free diet popularity cause a decline in bread consumption (9). Consumers may associate bread consumption with an increase in gastrointestinal symptoms (10). Their choice of bread is directly associated not only with their experiences with the product but also with consumers’ perceived health benefits of bread (11, 12). In previous studies in which consumers rated sensory descriptions of different bread samples without packaging (13, 14), dark brown colour, compact texture and sour flavour ratings were highly associated with perceived overall healthiness (14).

In recent years, sourdough bread has increased in popularity (13, 15). It may confer health benefits through the impact of the sourdough process on the nutritional content of the bread (15, 16). Sourdough bread has been shown to contain lower amount of FODMAPs than baker’s yeast bread and, hence, might be an alternative for people with gastrointestinal symptoms (10, 17). Presently, the impact of sourdough bread on gastrointestinal symptoms has only been investigated in a few studies, each with different results (18, 19).

To fill this knowledge gap, we conducted the health effects and consumer aspects of bread (HELFAB) study. The aim of the HELFAB-study was to investigate gut symptoms upon consuming sourdough bread compared to bread baked with yeast. Twenty healthy participants who were sceptical about consuming bread and/or having self-reported mild to moderate gastrointestinal symptoms upon consuming bread completed this randomised double-blind controlled cross-over study. This study lasted 5 weeks, commencing with a 2-week run-in (with baker’s yeast bread) before a 1-week intervention with sourdough or baker’s yeast, before crossing over after a 1-week wash-out (with baker’s yeast bread). Participants consumed a minimum of 200 g of bread per day. The participants did not know the kind of bread they consumed during the intervention. Qualitative research alongside randomised controlled trials (RCTs) can improve the understanding and effects of complex healthcare interventions (20). Thus, we interviewed 10 of the 20 participants in the HELFAB-study to gain more knowledge about their attitudes towards and experiences with sourdough and baker’s yeast bread before and after the intervention.

## Materials and methods

### Sampling and participants

This qualitative study was conducted amongst 10 out of 20 participants of the HELFAB-study. All of the participants of the HELFAB-study were recruited via the Oslo Metropolitan University (OsloMet) website and included students, employees and the public. OsloMet’s official social media sites, Facebook, Instagram and Snapchat, were used for recruitment. The website included information that we aimed to include participants who were sceptical towards bread. Participants contacted the project members. The process of recruiting started in August 2020 and continued throughout November of the same year. A more detailed description of the recruitment and study participants has been previously presented [Watters et al., to be submitted]. All 20 participants in the HELFAB-study were asked to participate in this qualitative sub-study.

### Data collection

Ten participants in the HELFAB-study were individually interviewed by the second author (master student in public health nutrition) prior to the intervention (in September 2020) and eight after the intervention (in December 2020). The interviews followed a semi-structured interview guide developed by the multi-professional project group. The second author pilot-tested the interview guide. The pilot test interview was included in the analysis, as only minor adjustments were made in the interview guide. Subsequent interviews were conducted by the second author, who did not have any personal relationships with the participants prior to the study. The interviews lasted from 20 to 35 min and were conducted via Zoom. The HELFAB-study was registered as a clinical trial (NCT04677881). Ethical approval for the experimental protocol of this study was obtained by the Regional Committees for Medical Research Ethics in South East Norway (Nr. 96264) and from the Norwegian Centre for Data Security (Nr. 382297). This study was in accordance with the Declaration of Helsinki. Participants gave their written informed consent to participate. Recruitment was carried out until we observed replication of responses with no new themes emerging from the interviews (21). We followed the consolidated criteria for reporting qualitative studies (COREQ) (22).

### Analysis

The interviews were audiotaped and transcribed by the second author. The first, second and the last authors read the transcripts. The first author, professor in health and nutrition communication and experienced in qualitative research, randomly compared some of the transcripts with the audiotapes to ensure the accuracy of the transcription process. The analysis was carried out by the first and second authors and was guided by thematic analysis, according to Braun and Clarke (23), and included the following steps: 1) becoming familiar with the data by repeated reading of each informant’s transcripts; 2) generating initial codes (words or short phrases in the transcripts) that were relevant to the research questions; 3) organising codes into sub-themes; 4) arranging sub-themes into overarching themes and 5) defining and naming the themes. The first and second authors conducted the analysis and discussed potential codes and themes with the last author. A qualitative software program, NVivo (12.0), was used to identify codes and systematise sub-themes.

## Results

Table 1 presents the background information of the participants. The participants were between 21 and 44 years old and resided in Oslo, Norway.

**Table 1:** Background information of the participants

Background information	Female (*n* = 8)	Male (*n* = 2)
Single household	3	2
Cohabitant without children	4	0
With children	2	0
Employed	6	1
Student	2	1

Table 2 summarises the sub-themes and main themes resulting from the data.

**Table 2:** Overview of main themes and sub-themes

Main themes	Sub-themes
Experiences with and attitudes towards bread prior to the RCT	Gastrointestinal problems related to bread consumption Improvement of problems with the stomach and intestine due to bread consumption Bread was associated with healthiness Easily available food group
Experiences with the intervention bread	No changes in problems in the stomach and intestine during the study Positive experiences with specific bread compared to previous problems in the stomach and intestine Problems in the stomach and intestine related to specific bread in the study Intervention bread associated with satiety Intervention bread experienced as less whole grain than the type usually consumed
Attitudes towards bread after the intervention	More positive attitude about bread due to the study Same attitude towards bread after the intervention
Motivation to participate in the RCT	Own health Gastrointestinal symptoms related to bread consumption Expectation that sourdough bread had a positive effect on their gastrointestinal symptoms

RCT, randomised controlled trial.

### Experiences with and attitudes towards bread prior to the RCT

Half of the interviewed participants experienced gastrointestinal symptoms, such as pain in the stomach, when they consumed bread prior to the RCT. They stated that these problems made them sceptical about eating bread. Other participants experienced that the consumption of dietary fibre solved their stomach problems. In this regard, participants often associated bread with healthiness, especially because of the content of fibre, as illustrated by the following statement by a female participant:

For me, it is important to eat whole grain bread, so that I get enough fibre… because if I do not get enough fibre, I get cramps in the stomach and obstipation. – participant 05

This participant said that she baked sourdough bread to increase the fibre content of her diet. None of the participants mentioned being aware of the use of FODMAPs to avoid their symptoms. Some participants preferred sourdough bread to baker’s yeast bread, since they experienced that sourdough bread was easier to digest. Other participants preferred sourdough bread, since they did not like the taste and smell of the yeast in yeast bread. Participants preferred homemade bread and expressed negative attitudes towards industrially produced bread due to uncertainty related to the healthiness of unknown ingredients. As illustrated in the following statement, the participants were concerned about the degree of bread processing:

I am more sceptical about bread from the grocery stores than from bakeries, (…) I think that bread from the grocery stores is processed. I try to avoid too processed foods because I want to know what I am eating. – participant 03

Participants were asked whether they were aware of the salt content in bread. The majority of the participants stated that they were not aware that bread contained a lot of salt, and that they did not check the salt content when buying bread, as illustrated by the following statement:

I have to admit that I do not think about salt when I buy bread, no. – participant 07.

Even though half of the participants had negative experiences with the consumption of bread, almost all of the participants considered bread to be an easily available food group that can fit every meal:

You can eat it [bread] for breakfast, lunch, dinner, and evening snack,… you can eat it with friends and when you are drinking wine. – participant 09

### Experiences with and attitudes towards the intervention bread

In general, the interviewed participants said that they did not make substantial changes to their daily meal routines during the study. Participants associated the study breads with increased satiety. They experienced that they had to eat so much bread that they could not eat anything else. Some were worried about not achieving the daily recommendations for other food classes, for example fruit and vegetables, owned to the satiety from bread:

‘I tried to eat vegetables; however, on the days when I had to eat 4 slices of bread together with my meal, I did not have any more space left for vegetables’. – participant 06.

Participants considered the intervention bread saturating; they experienced the breads in the study as less whole grain than the bread they usually ate. Many participants assumed that the increased amount of bread during the study would give them gastrointestinal problems; however, most of them did not recognise any changes during the intervention. Some participants were even surprised that they did not have any gastrointestinal problems during the intervention, as illustrated by the following statement:

I got very positively surprised when I ate so much bread, and I ate it every day, and did not get any problems in my stomach. I have not had any obstipation like I have had before. – participant 06

As mentioned above, the participants did not know the kind of intervention bread they consumed. However, some could identify sourdough bread due to its characteristic smell and taste. Participants often described the kind of bread they referred to:

My stomach was more bloated with yeast bread. My stomach felt more easy when I ate sourdough bread. Sometimes yeast bread felt like a stone in my stomach. – participant 05

Sourdough bread was often preferred due to taste, consistency and the feeling that it was easier to digest.

### Attitudes towards bread after the RCT

Interestingly, participants who were sceptical about bread prior to the study became more positive about bread because of their experiences with the intervention breads. This finding was mainly related to the taste and consistency of sourdough bread:

‘I started to want to try more sourdough bread because I liked it better. I’ve tried it before, but then I did not like it as much as the bread in the study. It was juicier, and not that sour’. – participant 02

Participants who were surprised that they did not have any gastrointestinal symptoms stated that they wished to continue to eat bread after the study:

I was very surprised. I did not experience any pain in my stomach. It seems that I actually tolerate bread, and now I want to include it into my daily diet again. – participant 03

Two participants had the same negative attitudes towards bread after the intervention as before due to experiences with abdominal pain, as illustrated by the experiences of a male participant:

‘The feeling that I had to visit the toilet immediately… this problem has gotten worse during the study’. – participant 09.

### Motivation to participate in the RCT

In the last interview, we asked the participants why they participated in the study. Engagement in their own health was the most common theme. The participants often wanted to find out why they experienced gastrointestinal problems related to bread consumption. They were very interested in the results of their biological tests to find out why they often experienced gastrointestinal challenges with bread. For instance, a male participant was curious to find out if sourdough bread could have beneficial effects on his gastrointestinal problems. Another participant said the following:

Some studies show that sourdough bread can have positive effects for those with high blood sugar, but not for all. So I would appreciate it if I could get to know if it makes any difference if I eat sourdough bread or not. – participant 07

## Discussion

Taken together, the intervention in this study lead to more positive attitudes towards bread amongst participants who stated to be sceptical about bread prior to the study. The participants described the bread during the intervention as both saturating and less whole grain than they usually eat.

Participants often preferred sourdough bread to baker’s yeast bread both prior to and after the study, since they experienced that sourdough bread was easier to digest. This finding is in line with clinical studies that found that sourdough-fermented breads are more digestible than breads baked with baker’s yeast alone (17). Interestingly, none of our participants seemed to be aware of FODMAPs. This is not in line with studies of patients with irritable bowel syndrome (IBS) (24, 25). For instance, 237 Polish patients with IBS had high levels of knowledge about low FODMAP diets, even though the respondents did not strictly comply with dietary guidelines. Age was significantly correlated with the respondents’ knowledge, and the participants’ familiarity with low FODMAP guidelines decreased with age (24). We did not assess whether our participants had received dietary information related to their symptoms. However, nutritional consultations about the health effects of FODMAPs did not significantly improve knowledge about the low-FODMAP diet amongst Polish patients in a previous study (24).

In the beginning of the intervention, half of the participants were sceptical about bread consumption due to gastrointestinal symptoms. Scepticism about bread is increasing worldwide, mainly due to the popularity of carbohydrate-restrictive diets (8). Even though many participants were sceptical about bread, they associated bread with healthiness. Participants in other studies also associated bread with healthiness (13, 14, 26, 27). One reason that participants in our study associated bread with healthiness might be the Norwegian food culture and reliance on dietary recommendations by health authorities that outline the consumption of whole-grain bread for a healthy diet (28). Other international studies have investigated consumers’ health-related perceptions of bread in general (12–14, 26, 27). Sandvik et al. investigated consumers’ health-related perceptions of bread by exploring which health-related quality attributes consumers associate with bread and whether there are differences with regard to age, gender and education level. The breads were perceived as healthy mainly because they ‘contain fibre’, are ‘good for the stomach’, have good ‘satiation’ and have beneficial ‘glycaemic properties’. Participants in our interviews were also concerned about eating enough fibre. A Polish cross-sectional study assessed whether consumers intended to eat bread enriched with fibre in the situation of the availability of plain bread and plain bread with grains. Participants with less education and lower incomes preferred to eat plain wheat rolls rather than rolls topped with sunflower seeds (26). Sajdakowska investigated whether the willingness to eat bread with health benefits is associated with individuals’ habits, taste and healthiness of bread. The results of the study showed that consumers who were more willing to eat bread with added fibre were those who paid more attention to health aspects, those who consumed more wholegrain bread and those who ate breads with grains more frequently (27).

To our knowledge, this is the first qualitative study investigating consumers’ experiences with sourdough bread compared to baker’s yeast bread. Several interviewed participants preferred the taste of sourdough bread compared to yeast bread. Other studies have found that taste is one of the major reasons for choosing bread. Participants in Sajdakowska’s study for whom taste was important were less willing to eat bread with reduced salt content compared with those who considered this attribute unimportant. In our study, the participants were not concerned about the salt content in bread (27).

### Study limitations

This study was conducted with a small sample size, which is typical of qualitative studies (29). Interviews were conducted via Zoom due to the COVID-19 pandemic. Even though face-to-face communication might have been of advantage, participants in another study described their interview experience as highly satisfactory and generally rated Zoom above alternative interviewing mediums, such as face-to-face and telephone (30).

## Conclusions

Participants associated the intervention bread with healthiness, as bread is an important source of dietary fibre. Sourdough bread with increased dietary fibre may be an important source of dietary fibre for those who perceive gastrointestinal problems from baker’s yeast bread. Thus, sourdough bread with dietary fibre may be an important component in a healthy diet.

## Credit author statement

LGH (PhD), MF, SJ, IR, CH (PhD), MCWM (PhD) and VHTH (PhD) designed the RCT. SINW and MG recruited the study participants. LGH had primary responsibility for the interview guide. MH conducted the interviews. LGH and MH analysed the interviews. VHTH contributed to the interpretation of the data. LGH drafted the manuscript. LGH and VHTH had primary responsibility for the final content. All authors read and approved the final manuscript.
